# Heteroepitaxial Cu_2_O thin film solar cell on metallic substrates

**DOI:** 10.1038/srep16272

**Published:** 2015-11-06

**Authors:** Sung Hun Wee, Po-Shun Huang, Jung-Kun Lee, Amit Goyal

**Affiliations:** 1 Materials Science and Technology Division, Oak Ridge National Laboratory, Oak Ridge, TN 37831, USA; 2 Department of Mechanical Engineering and Materials Science, University of Pittsburgh, Pittsburgh, PA15261, USA; 3 Research and Education in Energy, Environment & Water (RENEW), State University of New York (SUNY), Buffalo, NY 14260; 4 Department of Chemical & Biological Engineering State University of New York (SUNY), Buffalo, NY 14260; 5 Department of Materials Design & Innovation, State University of New York (SUNY), Buffalo, NY 14260; 6 Department of Electrical Engineering and Physics, State University of New York (SUNY), Buffalo, NY 14260

## Abstract

Heteroepitaxial, single-crystal-like Cu_2_O films on inexpensive, flexible, metallic substrates can potentially be used as absorber layers for fabrication of low-cost, high-performance, non-toxic, earth-abundant solar cells. Here, we report epitaxial growth of Cu_2_O films on low cost, flexible, textured metallic substrates. Cu_2_O films were deposited on the metallic templates via pulsed laser deposition under various processing conditions to study the influence of processing parameters on the structural and electronic properties of the films. It is found that pure, epitaxial Cu_2_O phase without any trace of CuO phase is only formed in a limited deposition window of P(O_2_) - temperature. The (00*l*) single-oriented, highly textured, Cu_2_O films deposited under optimum P(O_2_) - temperature conditions exhibit excellent electronic properties with carrier mobility in the range of 40–60 cm^2^ V^−1^ s^−1^ and carrier concentration over 10^16^ cm^−3^. The power conversion efficiency of 1.65% is demonstrated from a proof-of-concept Cu_2_O solar cell based on epitaxial Cu_2_O film prepared on the textured metal substrate.

Copper (I) oxide (Cuprous oxide, Cu_2_O) has a cubic structure with a lattice constant (a_0_) of 4.27 Å and is a native *p*-type semiconducting oxide due to negatively charged Cu vacancies that create an acceptor level ~0.16 eV above the valence band maximum^1^,^2^. It was the first semiconducting material discovered, and rectifier diodes based on this material were demonstrated as early as 1920s^3^. Cu_2_O has been regarded as one of the most promising semiconducting oxides available for photovoltaic (PV) application because it has several important characteristics such as a good absorption coefficient for visible light, a high mobility for the majority carriers (~100 cm^2^ V^−1^ s^−1^ at room temperature for a single crystal Cu_2_O), and a large minority carrier diffusion length (2–12 μm)^1^,^4^. More importantly, Cu_2_O is an earth abundant and non-toxic material^5^,^6^. For these reasons, even if its bandgap (E_g_ ~ 1.9–2.1 eV) at room temperature is relatively higher than the optimum value (E_g_ ~ 1.4–1.5 eV) for AM1.5 solar spectrum, Cu_2_O has been considered a material suitable for the realization of low cost and large scale PV devices production for several decades.

In Cu_2_O heterojunction cells, the majority of photons are absorbed by Cu_2_O layer and photo-generated holes are transported through Cu_2_O layer to a back electrode directly in contact with Cu_2_O layer, and electrons are transported through Cu_2_O layer to an *n*-type layer as minority and majority carriers, respectively. In order to fabricate high-performance Cu_2_O solar cells, it is vital to have excellent transport properties (carrier density and mobility, and minority carrier life time) inside Cu_2_O layer and at the junction interface. However, most Cu_2_O films have been prepared on un-textured or polycrystalline substrates by thermal oxidation, electro-deposition, and sputtering methods^1^,^4^,^7^,^8^,^9^,^10^,^11^,^12^,^13^,^14^,^15^,^16^,^17^,^18^,^19^,^20^. As a result, the films have a polycrystalline nature and rough surface morphologies, resulting in very defective films as well as poor interfaces at junctions and/or metal contacts comprising the cells. These defects act as sites for trapping, scattering, and recombination of the carriers, which decrease PV parameters including power conversion efficiency. Unlike defective polycrystalline films, single-crystal-like, epitaxial Cu_2_O films are expected to greatly reduce the defects by eliminating high angle grain boundaries which are major sources for defects generation and as a result, to have much better electronic and optical properties of the films. Several groups have reported epitaxial growth of Cu_2_O films with improved electronic properties via several deposition methods such as pulsed laser deposition (PLD), molecular beam epitaxy, and sputtering^21^,^22^,^23^,^24^,^25^. Matsuzaki *et al.*^22^ reported the improved majority carrier (hole) mobility from epitaxial Cu_2_O films grown via PLD. The mobility of 90 cm^2^ V^−1^ s^−1^ measured from the epitaxial Cu_2_O films is comparable to those of single crystals (~100 cm^2^ V^−1^ s^−1^) but is larger than the highest value (~60 cm^2^ V^−1^ s^−1^) reported from polycrystalline Cu_2_O films. However, epitaxial Cu_2_O films on rigid, size limited, expensive single crystal substrates are not practical for large-area, low-cost, Cu_2_O thin film based solar cells.

In this work, we report the first fabrication of heteroepitaxial Cu_2_O film solar cells on the textured, metallic foils. The Cu_2_O films prepared in optimum conditions exhibit exceptional transport properties, suitable for use as a *p*-type absorber layer of thin-film solar cells. Fabrication of high quality, epitaxial absorber layers on inexpensive, flexible, textured, metallic templates is potentially a very promising route to obtaining inexpensive, high-performance, non-toxic, earth-abundant solar cells^26^.

## Results

**Figure 1:**
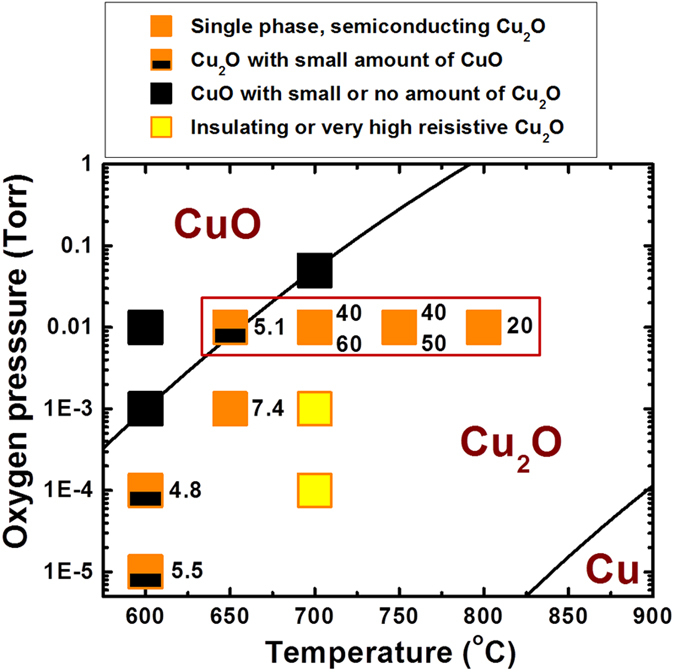
Phase and carrier mobility of the samples deposited at different P(O_2_) and temperatures.

**Figure 2:**
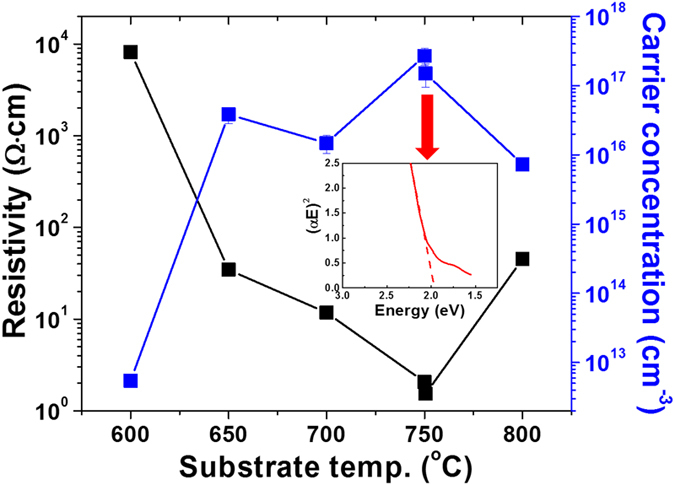
Resistivity and carrier concentration of Cu_2_O films grown at different deposition temperatures in a fixed P(O_2_) of 10 mTorr. Inset of the figure shows the optical absorption coefficient as a function of photon energy (=*hv*) as in equation, *αhv* = (*hv* − E_g_)^1/2^. Here, *h* is Planck’s constant and *v* is the frequency of the incident photon. Single phase Cu_2_O film grown at 750 °C was measured by a UV/V is spectrophotometer in a diffuse reflectance mode using an integrating sphere.

Considering a phase equilibrium diagram of copper-oxygen system^27^, Cu_2_O phase is thermodynamically stable only in limited oxygen partial pressure (P(O_2_)) - temperature range. Hence, it is vital to study the influence of P(O_2_) and deposition temperature (*T*_s_) on the phase, structural, and electronic properties of Cu_2_O films. Figure 1 summarizes the phase and carrier mobility of the films deposited in a wide range of P(O_2_) - *T*_s_ space. The samples deposited in P(O_2_) ~ 10 mTorr and T_s_ = 700–750 °C were confirmed to be bright-brown colored, single phase Cu_2_O films with the carrier mobility of 40–60 cm^2^ V^−1^ s^−1^. In addition to having such high carrier mobility, these films were measured to have high carrier concentrations of 10^16^–10^17^ cm^−3^ which consequently, lead to low resistivity (ρ) of ~10 Ω · cm, as shown in Fig. 2. It is also confirmed that the single phase Cu_2_O films have the bandgap (E_g_) of ~2.0 eV, close to E_g_ of 1.9–2.1 eV typically reported from Cu_2_O^1^,^4^, as shown in the inset of Fig. 2. Since the Cu_2_O is a direct band-gap semiconductor, the optical band gap (*E*_*g*_) was obtained from the plot of the optical absorption coefficient as a function of photon energy (=*hv*) as in equation, *αhv* = (*hv* − E_g_)^1/2^. When the films were deposited outside this optimum P(O_2_) - *T*_s_ range but still in P(O_2_) - *T*_s_ range where Cu_2_O phase is thermodynamically stable, the samples exhibit either (or both) degraded structural or (and) electronic properties. For instance, the films deposited at 600 °C in P(O_2_) = 0.01–0.1 mTorr were observed to have dark-brown color due to the presence of small amount of black-colored CuO phase with Cu_2_O phase in the films which was also confirmed by X-ray diffraction (XRD) analysis. The films deposited in P(O_2_) - *T*_s_ regions near Cu_2_O/CuO phase boundary line also have dark-brown or black color due to CuO phase formed partially or mostly in the films. Formation of CuO phase in these films, because of its poor conductivity^28^, is a primary reason that these films exhibit an insulating behavior or poor electronic performance with low carrier mobility (4.8–5.5 cm^2^ V^−1^ s^−1^) and concentration less than 1 × 10^14^ cm^−3^. On the other hand, the films grown in low P(O_2_) = 0.1–1 mTorr at 700 °C also exhibit an insulating behavior with extremely high resistivity even though XRD results confirm that the films are highly epitaxial and composed of single Cu_2_O phase with no 2^nd^ phases including CuO phase. In this case, poor conductivity for the films should be caused by charge compensation of negatively charged copper vacancies via positively charged oxygen vacancies that should form and increase in their density when the films were deposited in lower P(O_2_) and thereby, both concentration and mobility of holes were substantially suppressed^29^.

**Figure 3:**
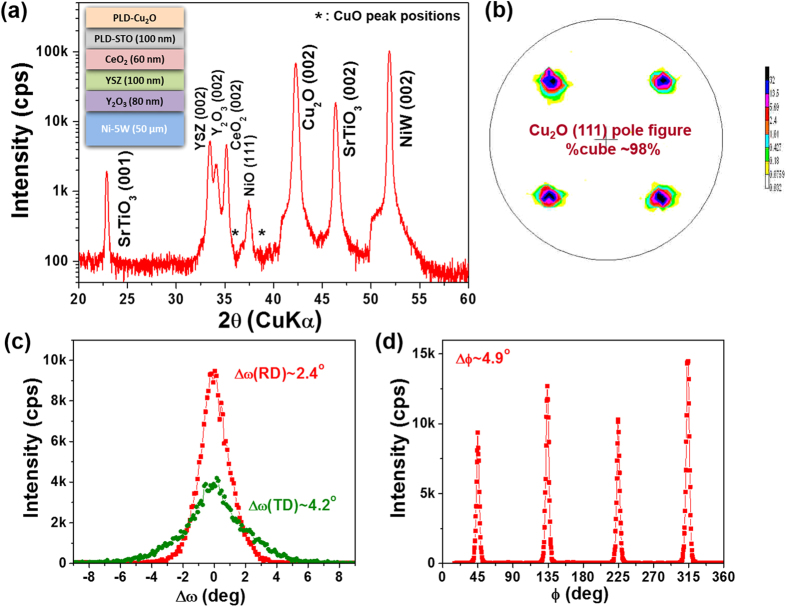
(**a**) θ–2θ XRD scan result for ~0.5-μm-thick Cu_2_O film grown on the Ni-W substrates with oxide buffer layers – Cu_2_O/STO/CeO_2_/YSZ/Y_2_O_3_/NiW (growth condition: P(O_2_) = 10 mTorr and *T*_s_ = 750 °C). Inset of the figure schematically illustrates the multilayer architecture for epitaxial growth of Cu_2_O layer on the Ni-W metallic template. (**b**) (111) pole figure, (**c**) (002) ω-scans for both rolling (φ = 0°) and transverse (φ = 90°) directions, and (**d**) (111) φ-scan for the Cu_2_O film indicating that the films are highly cube-textured with small Δω and Δφ values.

Figure 3 summarizes XRD results of a Cu_2_O film grown on the NiW template with the developed oxide buffer architecture as illustrated in the inset of Fig. 3a in an optimized condition of P(O_2_) = 10 mTorr and T_s_ = 750 °C. The θ–2θ scan XRD result shown in Fig. 3a shows strong (00*l*) peak intensities from buffer layers and Cu_2_O film, indicating that all the layers are highly out-of-plane textured. Although no peaks related to CuO phase or other Cu_2_O orientations are visible, small NiO (111) peak at 2θ ~37.5° can be seen due to the oxidation of NiW metallic substrate during Cu_2_O growth. The self-limiting oxidation of NiW substrate forming a very thin, continuous, smooth NiO layer between NiW substrate and Y_2_O_3_ seed layer was commonly observed during film deposition in oxygen atmosphere^30^,^31^. Figure 3b–d show (002) ω- and (111) φ-scans as well as (111) pole figure for Cu_2_O layer. Small full-width-half-maximum values (Δω and Δφ) of ω- and φ-scans around 2.4° and 4.9° as well as a clear four-fold symmetry with 94% of cube texture suggest that the Cu_2_O film has excellent cube-on-cube epitaxy with the buffer layers and the substrate. Table 1 shows a summary of Δω, Δφ, and %cube texture for all layers from the starting Ni-W substrate up to Cu_2_O layer. The oxide buffer layers have better out-of-plane texture with smaller Δω values in both rolling (φ = 0°) and transverse (φ = 90°) directions than the Ni-W substrate, while they have similar in-plane texture determined by the Δφ value. Improving the out-of-plane texture by the deposition of an initial oxide seed layer (e.g. Y_2_O_3_) layer on the Ni-W substrate has been reported and was explained on the basis of crystallographic tilting of the epitaxial film^32^. It is also observed that the in-plane and out-of-plane textures of oxide buffer layers are further improved by PLD of SrTiO_3_ (STO) top layer which exhibits ~0.8–1.4° smaller Δω and Δφ values than the underneath CeO_2_ layer. Such improved textures of the top layer lead Cu_2_O films to have smaller Δω and Δφ values and thereby, smaller angles of grain boundaries in the layer, as compared to the bare Ni-W substrate. It is noted that the basal planes of Y_2_O_3_, YSZ, and CeO_2_ are 45° rotated relative to the basal planes of CuO_2_, STO, and Ni-W because of the smaller lattice mismatches between [110]_Y2O3,YSZ,CeO2_ and [100]_Cu2O,STO,Ni−W_.

**Figure 4:**
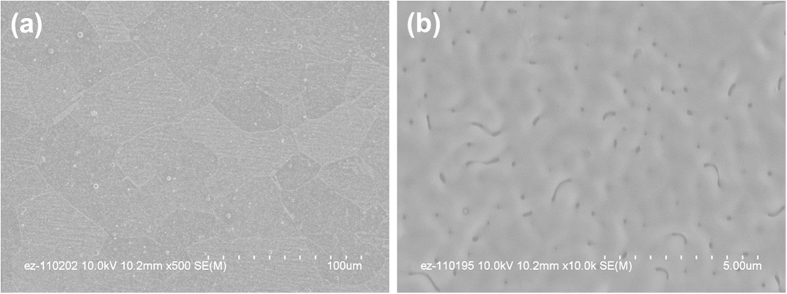
Plan-view SEM images for the Cu_2_O film grown on the Ni-W substrates. (**a**) Low magnification SEM image showing the grain structures with large grain sizes of 50–100 μm and (**b**) High magnification SEM image showing dense and smooth surface morphological features of the film.

Field-emission scanning electron microscope (FE-SEM) images in Fig. 4 show microstructural features of highly textured Cu_2_O films deposited on the metallic templates. Low magnification plan-view image in Fig. 4a shows that the Cu_2_O layer is very dense and replicates the grain structures with grain sizes of 50–100 μm, initially developed from biaxially-textured, cube-oriented NiW substrates^33^,^34^. The high magnification image in Fig. 4b shows that the film has a smooth surface morphology, but there are a few of surface pores. The dominant growth mechanism of Cu_2_O films on STO should be either three-dimensional (3D) island (Volmer-Weber, V-W)^24^,^35^ or two-dimensional layer-by-layer followed by 3D island (Stranski-Krastanov, S-K) growth modes considering their surface energy (γ_STO_ ~ 0.8 ~ 1.6 J/m^2^, γ_Cu2O_ ~ 0.8–1.7 J/m^2^)^36^,^37^ and lattice mismatch (~8.6%). So, the surface pores could be caused by incomplete coalescence during film growth via V-W or S-K growth mechanism with increasing the film thickness.

**Figure 5:**
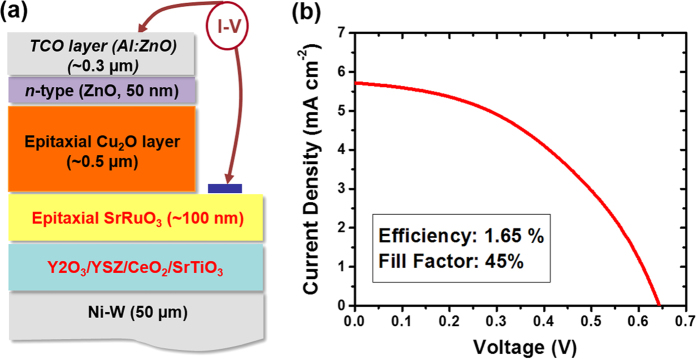
(**a**) Schematic illustration and (**b**) Current-voltage curve under simulated AM 1.5 illumination for a solar cell device based on epitaxial Cu_2_O layer on the NiW metallic template.

Figure 5 shows a schematic of the solar cell structure with ~0.5 μm thick, epitaxial Cu_2_O absorber layer on NiW foil with the developed buffer architecture and the results of current-voltage (*I*-*V*) measurement under simulated AM1.5 illumination. For the fabrication of *p*-*n* junction devices with epitaxial Cu_2_O films, additional layers that act as a bottom electrode, an *n*-type layer, and a top electrode were deposited underneath and on top of Cu_2_O *p*-type absorber layer, as illustrated in Fig. 5a. As a bottom electrode, we employ SrRuO_3_ (SRO) conductive oxide because of its low resistivity (ρ = ~2 × 10^−4^ Ω · cm) and a suitable work function (φ_SRO_ ~ 5.2 eV) to make Ohmic contact with Cu_2_O layer^4^. Moreover, SRO has good structural compatibility with STO and CuO_2_ that enables epitaxial growth of both Cu_2_O and SRO films on STO underlayer. We also experimentally confirm that insertion of SRO electrode between Cu_2_O films and STO layer does not change the epitaxial quality and microstructure of Cu_2_O films using XRD and SEM characterizations. As an *n*-type layer, ZnO layer is selected since the layer has been considered the best *n*-type layer currently available^8^,^9^,^10^,^14^,^15^,^18^,^19^. Finally, as a top electrode layer, Al-doped ZnO, transparent conductive oxide (TCO) layer was deposited on top of *n*-type ZnO layer due to its excellent structural and chemical compatibility with *n*-type ZnO layer compared to ITO layer, another popular TCO layer for Cu_2_O solar cells^7^. The device shows power conversion efficiency of 1.65%, with an open circuit voltage (V_OC_) of 0.64 V, a short circuit current (J_SC_) of 5.7 mA cm^−2^, and a fill factor (FF) of 45%.

## Discussion

It is essential for the samples to have both high carrier mobility and concentration to be used as an absorber layer. The samples with high carrier concentration but low mobility are most likely to be very defective with a high density of chemical imperfections or defects that act as recombination centers. On the other hand, the samples with high carrier mobility but low carrier concentration are typically too resistive and their Fermi energy level is not suitable for building a high built-in potential at the interface of *p*-*n* junctions. Both cases should limit significantly physical properties related to solar power conversion efficiency. Although some previous efforts have demonstrated Cu_2_O films with either high carrier mobility (over 50 cm^2^ V^−1^ s^−1^) or high carrier concentration (over 10^16^ cm^−3^), deposition of Cu_2_O films having both high carrier mobility and concentration without using single crystalline substrates has been considered very challenging in the field. To our best knowledge, a recent work only reported high carrier mobility of 50–70 cm^2^ V^−1^ s^−1^ as well as high concentration of ~1 × 10^16^ cm^−3^ from very thin (70 nm thick) epitaxial Cu_2_O films on (100) MgO single crystal substrates^23^, similar to the values reported in this study on technical substrates that can be scaled to very large-areas at low-cost.

The power conversion efficiency of 1.65% achieved in this study is comparable to most Cu_2_O solar cells (0.1–2%) prepared by thermal oxidation of Cu foils and thin film deposition techniques such as electrodeposition and sputtering^4^,^7^,^8^,^9^,^10^,^11^,^15^,^18^. We fitted J-V curve of the solar cell using a well-known equivalent circuit model where series resistance (R_S_) and shunt resistance (R_SH_) are connected to the solar cell composed of an electric current source and a *p*-*n* junction diode. Fitting results show that R_S_ and R_SH_ are 5 Ω · cm^−2^ and 600 Ω · cm^−2^, respectively. This indicates that Cu_2_O solar cell in this study does not follow an ideal behavior of solar cells composed of an electric current source and a diode. The carrier recombination in Cu_2_O film and at Cu_2_O/ZnO interface and the series resistance of PLD grown films are responsible for a gap between the theoretical behavior and real behavior of Cu_2_O solar cell. Although the power conversion efficiencies of 4–6.1% were recently reported from the solar cell fabricated on thermally oxidized, thick Cu_2_O sheets^14^,^20^, our results indicate that thin film Cu_2_O solar cells can be successfully implemented onto NiW foil templates. Higher efficiency of the cell would be expected with further optimization of Cu_2_O absorber layer thickness and cell fabrication process to improve light absorption. In addition, we will also explore ways, such as hydrogenating thin ZnO layer to passivate Cu_2_O/ZnO interface and finding more appropriate *n*-type oxide materials having better structural and chemical compatibility with Cu_2_O, to minimize the carrier recombination at Cu_2_O/ZnO interface and ultimately improve the power conversion efficiency of the solar cells.

In conclusion, we demonstrate heteroepitaxial thin film Cu_2_O solar cells on low-cost, flexible, NiW foil templates. The study reveals an optimum P(O_2_) - temperature region where the films have only single oriented, pure Cu_2_O phase without the presence of high resistive CuO phase and as a result, excellent electronic properties with high carrier mobility in the range of 40–60 cm^2^ V^−1^ s^−1^ and concentration over 10^16^ cm^−3^. Detailed XRD analysis confirms that the films have excellent cube-on-cube epitaxy with over 98% cube texture and much smaller Δω and Δφ values than those of buffers and NiW metallic substrate. As a proof of concept, the power conversion efficiency of 1.65% is achieved for a cell fabricated on 0.5 μm thick, epitaxial Cu_2_O absorber layer on the buffered NiW template.

## Methods

Biaxially-textured Ni-W foils with three heteroepitaxial oxide buffers comprised of CeO_2_ cap (60 nm)/YSZ barrier (100 nm)/Y_2_O_3_ seed (80 nm) grown by reactive sputtering were used^32^,^33^. This specific template architecture was developed for epitaxial growth of YBa_2_Cu_3_O_7−x_ superconducting films capable of carrying high critical currents, and such templates are routinely fabricated in lengths of 100’s of meters in a roll-to-roll configuration^33^,^34^. To complete the buffer stack for epitaxial Cu_2_O films, ~100 nm thick, (100) oriented, epitaxial STO layer was then grown via PLD using a KrF excimer laser (248 nm) at *T*_s_ of 700 °C and P(O_2_) of 10 mTorr. Our preliminary study confirmed that STO layer was required for epitaxial growth of Cu_2_O films because of poor chemical compatibility between CeO_2_ and Cu_2_O layers. All Cu_2_O films with ~0.5 μm thickness were deposited in a wide P(O_2_) - *T*_s_ range by PLD at optimized laser energy density and target-substrate distance which were ~3 J cm^−2^ and 5 cm, respectively. To fabricate a solar cell structure with an epitaxial Cu_2_O absorber layer, we additionally introduced ~100 nm thick epitaxial SRO conductive oxide layer between Cu_2_O and STO layers as well as *n*-type ZnO and Al-doped ZnO TCO layer on top of Cu_2_O layer. The crystalline phase, texture, and microstructural properties of the samples were characterized by using XRD and FE-SEM. Electronic properties (resistivity, carrier mobility, and concentration) of Cu_2_O films were characterized at Van der Pauw geometry using Hall effect measurement. Optical properties of the films were measured by a UV/Vis spectrophotometer. Photovoltaic properties were measured under AM 1.5 G simulated sunlight (PV Measurements, Inc.) with the aid of an electrochemical workstation (CH Instruments, CHI 660C). The active cell size was 6 mm^2^ and we used a mask during illumination to minimize the effect of the peripheral illumination on energy conversion efficiency measurements.

## Additional Information

**How to cite this article**: Wee, S. H. *et al.* Heteroepitaxial Cu_2_O thin film solar cell on metallic substrates. *Sci. Rep.* **5**, 16272; doi: 10.1038/srep16272 (2015).

## Figures and Tables

**Table 1:** FWHM values (Δω and Δφ) of ω-, φ-scans, and % cube textures for the sample that includes Cu_2_O (~0.5 μm)/SrTiO_3_ (100 nm)/CeO_2_ (60 nm)/YSZ (100 nm)/Y_2_O_3_ (80 nm)/Ni - 5 at% W (50 μm).

Layer (lattice parameter, a_0_)	Δω (002 or 004)		Δφ (111)	%cube
	(φ = 0°)	(φ = 90°)*		
Cu_2_O (4.27 Å)	2.4°	4.7°	4.9°	98%
SrTiO_3_ (3.91 Å)	2.8°	5.0°	5.4°	95%
CeO_2_ (5.41 Å)	3.7°	6.4°	6.2°	97%
YSZ (5.15 Å)	3.9°	6.5°	6.1°	97%
Y_2_O_3_ (5.30 Å)	3.9°	6.5°	6.1°	94%
Ni - 5 at% W (3.52 Å)	5.2°	9.4°	6.3°	99%

^*^φ = 0° and = 90° represent rolling and transverse directions of Ni-W tape, respectively.

## References

[b1] Rai B. P. Cu_2_O solar cells: a review. Solar Cells 25, 265 (1988).

[b2] Scanlon D. O. & Watson G. W. Undoped n-type Cu_2_O: fact or fiction? J. Phys. Chem. Lett. 1, 2582 (2010).

[b3] Grondahl L. O. The copper-cuprous-oxide rectifier and photoelectric cell. Reviews of Modern Physics 5, 141 (1933).

[b4] Olsen L. C., Addis F. W. & Miller, W. Experimental and theoretical studies of Cu_2_O solar cells. Solar Cells 7, 247 (1982).

[b5] Wadia C., Alivisatos, A. P. & Kammen D. M. Materials availability expands the opportunity for large-scale photovoltaics deployment. Environ. Sci. Technol. 43, 2072 (2009). 19368216 10.1021/es8019534

[b6] Green M. A. Consolidation of thin-film photovoltaic technology: the coming decade of opportunity. Prog. Photovolt. Res. Appl. 14, 383 (2006).

[b7] Mittiga A., Salza E., Sarto F., Tucci M. & Vasanthi R. Heterojunction solar cell with 2% efficiency based on a Cu_2_O substrate. Appl. Phys. Lett. 88, 163502 (2006).

[b8] Izaki M., Shinagawa T., Mizumo K. T., Ida Y., Inaba M. & Tasaka A. Electrochemically constructed p-Cu_2_O/n-ZnO heterojunction diode for photovoltaic device. J. Phys. D: Appl. Phys. 40, 3326 (2007).

[b9] Akimoto K. *et al.* Thin film deposition of Cu_2_O and application for solar cells. Sol. Energy 80, 715 (2006).

[b10] Herion J., Niekisch E. A. & Sharl G. Investigation of metal oxide/cuprous oxide heterojunction solar cells. Sol. Energy Materials 4, 101 (1980).

[b11] Katayama J., Ito K., Matsuoka M. & Tamaki J. Performance of Cu_2_O/ZnO solar cell prepared by two-step electrodeposition. J. Appl. Electrochem. 34, 687–692 (2004).

[b12] Cui J. & Gibson U. J. A simple two-step electrodeposition of Cu_2_O/ZnO nanopillar solar cells. J. Phys. Chem. C 114, 6408–6412 (2010).

[b13] Yuhas B. D. & Yang P. Nanowire-based All-Oxide Solar Cells. J. Am. Chem. Soc. 131, 3756–3761 (2009). 19275263 10.1021/ja8095575

[b14] Minami T., Nishi Y., Miyata T. & Nomoto J. I. High-efficiency Oxide Solar Cells with ZnO/Cu_2_O Heterojunction Fabricated on Thermally Oxidized Cu_2_O Sheets. Appl. Phys. Exp. 406, 1253 (2011).

[b15] Minami T., Tanaka H., Shimakawa T., Miyata T. & Sato H. High-efficiency Oxide Heterojunction Solar Cells using Cu_2_O sheet. Jpn. J. Appl. Phys. 43, L917 (2004).

[b16] Motoyoshi R. *et al.* Structure and photovoltaic activity of cupric oxide-based thin film solar cells. J. Ceram. Soc. Jpn. 118, 1021 (2010).

[b17] Han K. & Tao M. Electrochemically deposited p-n homojunction cuprous oxide solar cells. Sol. Energy Mater. Sol. Cells 93, 153 (2009).

[b18] Tanaka H. *et al.* Electrical and optical properties of TCO–Cu_2_O heterojunction devices. Thin Solid Films 469–470, 80 (2004).

[b19] Chou S. M., Hon M. H., Leu I. C. & Lee Y. H. Al-Doped ZnO/Cu_2_O Heterojunction Fabricated on (200) and (111)-Orientated Cu_2_O Substrates. J. Electrochemical Soc. 151, H923 (2008).

[b20] Minami T., Nishi Y. & Miyata T. H. Heterojunction solar cell with 6% efficiency based on an n-type aluminum–gallium–oxide thin film and p-type sodium-doped Cu_2_O sheet. Appl. Phys. Exp. 8, 022301 (2015).

[b21] Ogale S. B., Bilurkar P. G. & Mate N. Deposition of epitaxial Cu_2_O films on (100) MgO by laser ablation and their processing using ion beams. J. Crystal Growth 128, 714 (1993).

[b22] Matsuzaki K. *et al.* Epitaxial growth of high mobility Cu_2_O thin films and application to p-channel thin film transistor. Appl. Phys. Lett. 93, 202107 (2008).

[b23] Darvish D. S. & Atwater, H. A. Epitaxial growth of Cu_2_O and ZnO/Cu_2_O thin films on MgO by plasma-assisted molecular beam epitaxy. J. Crystal Growth 319, 39 (2011).

[b24] Lee S., Liang C. W. & Martin L. W. Synthesis, control, and characterization of surface properties of Cu_2_O nanostructures. ACS Nano 5, 3736 (2011). 21486071 10.1021/nn2001933

[b25] Yin Z. G. *et al.* Two-dimensional growth of continuous Cu_2_O thin films by magnetron sputtering. Appl. Phys. Lett. 86, 061901 (2005).

[b26] Wee S. H. *et al.* Heteroepitaxial film silicon solar cell grown on Ni-W foils. Energy & Environmental Science 5, 6052 (2012).

[b27] Ito T., Yamaguchi H., Okabe K. & Masumi T. Single-crystal growth and characterization of Cu_2_O and CuO. J. Mater. Sci. 33, 3555 (1998).

[b28] Valladares L. *et al.* Crystallization and electrical resistivity of Cu_2_O and CuO obtained by thermal oxidation of Cu thin films on SiO_2_/Si substrates. Thin Solid Films 520, 6368 (2012).

[b29] Li J. *et al.* Probing defects in Nitrogen-Doped Cu_2_O. Sci. Rep. 4, 7240, 10.1038/srep07240 (2014). 25430516 PMC5384195

[b30] Wee S. H., Goyal A., Martin P. M. & Heatherly L. High in-field critical current densities in epitaxial NdBa_2_Cu_3_O_7−δ_ thin films on RABiTS by pulsed laser deposition. Supercond. Sci. Technol. 19, 865 (2006).

[b31] Leonard K. J. *et al.* Identification of a self-limiting reaction layer in Ni-3 at% W rolling-assisted biaxially textured substrates. Supercond. Sci. Technol. 17, 1295 (2006).

[b32] Cantoni C. *et al.* Influence of oxygen deficiency on the out-of-plane tilt of epitaxial Y_2_O_3_ films on Ni-5% W tapes. J. Mater. Res. 24, 520 (2009).

[b33] Goyal A. *et al.* Using RABiTS to fabricate high-temperature superconducting wire. JOM 51, 19 (1999).

[b34] Rupich M. W. *et al.* Advances in second generation high temperature superconducting wire manufacturing and R&D at American Superconductor Corporation. Supercond. Sci. Tech. 23, 014015 (2010).

[b35] Lyubinetsky I., Lea A. S., Thevuthasan S. & Baer, D. R. Formation of epitaxial oxide nanodots on oxide substrate: Cu_2_O on SrTiO_3_(100). Surf. Sci. 589, 120 (2005).

[b36] Zemzemi M. & Alaya S. First principles study of the structural and electronic properties of the ZnO/Cu_2_O Heterojunction. Materials Sciences and Applications 6, 661 (2015).

[b37] Sano T., Saylor D. M. & Rohrer G. S. Surface energy anisotropy of SrTiO_3_ at 1400 °C in Air. J. Am. Ceram. Soc. 86, 1933 (2003).

